# A case of a jejunal gastrointestinal stromal tumor with significantly elevated CA19-9 levels

**DOI:** 10.1016/j.ijscr.2020.06.018

**Published:** 2020-06-12

**Authors:** Koichi Mohri, Kazuhiro Hiramatsu, Yoshihisa Shibata, Taro Aoba, Masahiro Fujii, Atsuki Arimoto, Akira Ito, Takehito Kato

**Affiliations:** Department of General Surgery, Toyohashi Municipal Hospital, Toyohashi, Aichi, Japan

**Keywords:** GIST, gastrointestinal stromal tumor, CA19-9, carbohydrate antigen 19-9, CT, computed tomography, CEA, carcinoembryonic antigen, Tumor marker, Carbohydrate antigen 19-9, Gastrointestinal stromal tumour

## Abstract

**Introduction:**

A gastrointestinal stromal tumor (GIST) with an elevated serum tumor marker level is very rare. We report a case of jejunal GIST associated with extremely elevated levels of serum carbohydrate antigen 19-9 (CA19-9).

**Presentation of case:**

A 61-year-old woman was referred to our hospital for examination of an abdominal tumor. Laboratory tests revealed extremely elevated CA19-9 levels (13,498 U/mL). Enhanced abdominal computed tomography demonstrated a well-enhanced, round 40 mm tumor. The patient underwent a jejunectomy and lymph node dissection. Based on the postoperative pathological findings, the tumor was diagnosed as a GIST. Microscopically, a solid region of the resected tumor showed negative staining for CA19-9. The serum CA19-9 level drastically decreased postoperatively.

**Discussion:**

Increased proliferation of epithelial cells secondary to inflammation and ulceration of epithelia may lead to increased secretion and accumulation of CA19-9, which is consequently released into the blood circulation.

**Conclusion:**

In cases of GIST, an isolated increase of serum CA19–9 is extremely rare; but they are not necessarily associated with malignant transformation.

## Highlights

• We report a jejunal GIST with elevated CA19-9 levels that normalized after resection.
• A correlation between GIST and the elevation of CA19-9 has been unknown.
• The mechanism might be inflammatory hypersecretion by normal epithelial cells.
• Careful interpretation of elevated CA19-9 is mandatory.
• The possible surgical procedures should not be limited to a minimally-invasive approach.

## 1 Introduction

Carbohydrate antigen 19-9 (CA19-9) is a tumor-associated antigen that is elevated in many types of cancer. A gastrointestinal stromal tumor (GIST) with an elevated CA19-9 level is very rare. We report a case of jejunal GIST associated with an extremely elevated level of serum CA19-9. This case report has been written in line with the SCARE criteria [1].

## 2 Presentation of case

**Fig. 1:**
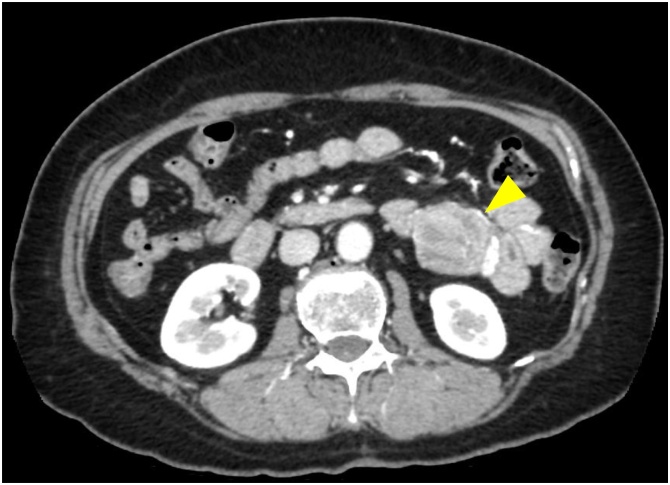
A contrast-enhanced CT scan shows a 40 mm well-enhanced, round tumor that was located at the jejunum (arrow head).

**Fig. 2:**
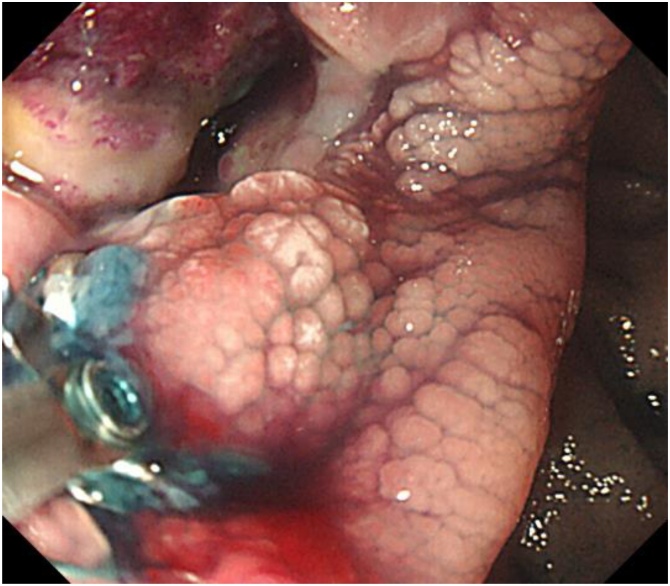
A single balloon enteroscopy revealed a large submucosal tumor with a central deep ulceration.

A 61-year-old woman underwent trans-abdominal ultrasonography for chronic back pain, which revealed a round tumor near the pancreatic tail. She was referred to our hospital for further examination. She had no past medical or surgical history. On physical examination, the mass was not palpated. Laboratory findings indicated that the CA19-9 level was significantly elevated (13,498 U/mL). The carcinoembryonic antigen (CEA) level was within the normal limit. A contrast-enhanced computed tomography (CT) demonstrated a 40 mm well-enhanced, round tumor that was located at the jejunum (Fig. 1). Double-contrast gastrography showed an irregular lesion protruding into the wall at the proximal jejunum 20 cm distal to the side of the Treiz ligament. Single-balloon enteroscopy revealed a large submucosal tumor with a deep central ulceration (Fig. 2). The ulcerative lesion bled easily and had some exposed vessels. Histological examination of a forceps biopsy sample was performed, but no pathological diagnosis could be achieved preoperatively.

It was suggested this tumor might have malignant characteristics; therefore, primary jejunal cancer, malignant lymphoma, aberrant pancreatic cancer, and GIST were suggested by the differential diagnosis. Laparotomy showed a tumor covered on the smooth surface at the distal side of the Treiz ligament. No invasion to the surrounding organs was detected. The patient underwent partial jejunectomy and lymph node dissection was performed.

**Fig. 3:**
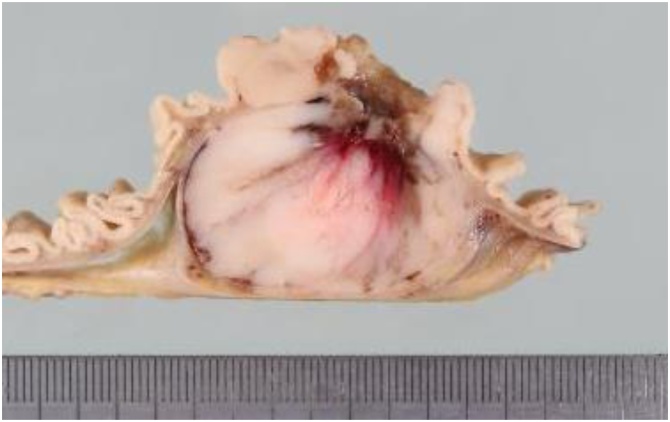
The cut section of the gross specimen shows an intramural mass, which measures 40 × 40 mm. A depressed ulcerative lesion is identified.

**Fig. 4:**
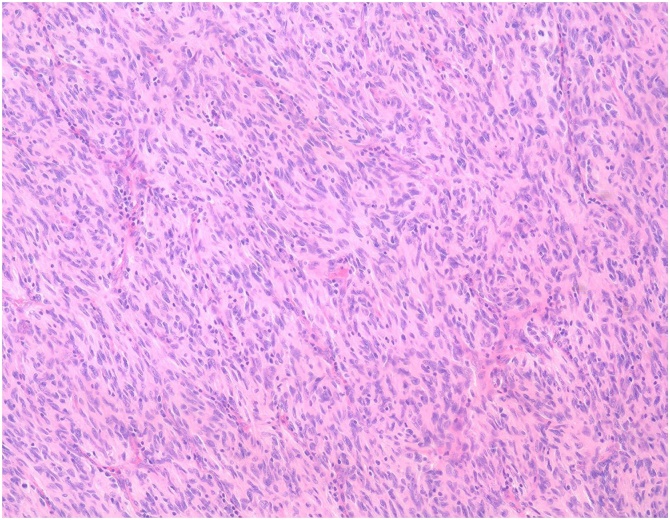
Microscopic findings show that spindle cells with dense nuclei proliferate intricately, forming short fascicles.

**Fig. 5:**
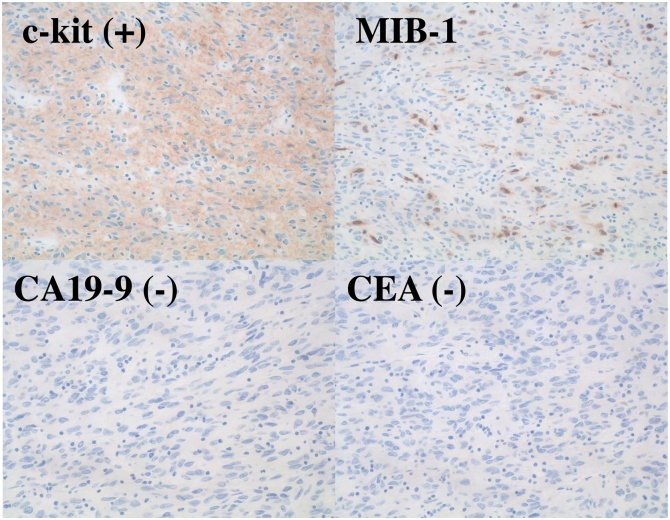
Immunohistochemically, the tumor was strongly positive for c-kit and negative for CA19-9 and CEA. The MIB-1 labeling index was <10 %.

Macroscopically, the tumor formed a 40 × 40 mm white-colored homogenous mass (Fig. 3). The tumor was located in the submucosal layer of the intestinal wall with deep ulceration on the mucosal surface. Microscopically, a solid region of the resected tumor showed a spindle-cell appearance with positive staining for c-kit and negative staining for CD34, CEA, and CA19-9 (Fig. 4). The diagnosis was primary GIST of the small intestine. No lymph node metastasis was detected. The mitotic count was 30/50 high-power fields (HPFs) and the MIB-1 labeling index was <10 % (Fig. 5). The GIST was classified in the high-risk group based on modified Fletcher’s classification and Miettinen’s classification.

No postoperative events occurred, and the patient was discharged on postoperative day 7. After surgery, the level of serum CA19-9 decreased to normal within limits. There were no signs of recurrence 26 months postoperatively.

## 3 Discussion

It is assumed that GISTs originate from the interstitial cells of Cajal stem cells within the wall of the gastrointestinal tract; therefore GISTs arising in the gastrointestinal tract are typically found in a subepithelial location [2]. In addition, large tumors may have an epithelial ulceration. GISTs are frequently found in the stomach (40%–60%), followed by the small bowel (25 %) [3]. Jejunal GISTs account for 10 % of GISTs arising from the gastrointestinal tract [4]. CT scan typically demonstrates GISTs as a heterogeneous occupied lesion with high vascularity [5]. The preoperative diagnosis may be challenging and the definite diagnosis is usually derived from a postoperative pathological examination.

CA19-9 was shown to be a tumor-associated antigen elevated in the blood of many patients with pancreatic cancers, cancers of the upper gastrointestinal tract, ovarian cancer, hepatocellular cancer and colorectal cancer [6,7]. Furthermore, serum CA19-9 levels are also elevated in various benign conditions including diabetes, diverticulitis, interstitial pulmonary disease, and obstructive jaundice [[8], [9], [10], [11]].

Regarding serum tumor markers of GISTs, none are expected to be positive due to the mesenchymal origin of the tumor. However, there were several reports from Japan that serum CEA and/or CA19-9 level were elevated in cases of GIST, and were normalized after resection in all cases [[12], [13], [14], [15]]. In this case, the patient had a CA19-9 level of >13,000 U/mL, and the preoperative diagnosis was not obtained. In cases in which the jejunal tumor was pathologically diagnosed as GIST preoperatively, a less-invasive laparoscopic approach was selected. The laparotomy approach and lymphadenectomy are implemented in consideration for malignancy.

In this case, a solid region of the resected tumor showed negative staining for CA19-9 microscopically and the serum CA19-9 level decreased postoperatively. A correlation between the clinical features of GIST and the elevation of serum tumor markers has been unknown. The underlying mechanism may be an inflammatory hypersecretion of CA19-9 by normal epithelial cells. Small concentrations of CA19-9 have been immunohistologically localized on the epithelia of adult gastrointestinal tract [16]. Furthermore, it was reported that CA19-9 was histopathologically positive in ulceration of the overlying normal mucosa secondary to GIST [14]. A report suggested that benign broncho-pulmonary changes, such as inflammation and bronchiectasis, promoted considerable production of CA19-9 in respiratory bronchioles [17]. Immunohistochemical analysis has shown high CA19-9 reactivity in inflammatory areas [18]. Thus, it is hypothesized that increased proliferation of epithelial cells secondary to irritation, inflammation, and ulceration of the epithelia leads to an increased secretion and accumulation of CA19-9, which is consequently released into the blood circulation.

## 4 Conclusion

Cases of GIST with an isolated increase of serum CA19-9 are extremely rare, but are not necessarily associated with malignant transformation. However, when malignancy cannot be ruled out, careful interpretation is mandatory. Therefore, the possible surgical procedures should not be limited to a minimally-invasive approach.

## Declaration of Competing Interest

The authors declare that they have no conflicts of interest.

## Sources of funding

There are no study sponsors or sources of funding.

## Ethical approval

Hospital Ethics Committee and Institutional Review Board of Toyohashi municipal hospital exempt ethical approval for a case report involving a single patient as far as written informed consent was obtained from patient for publication.

## Consent

Written informed consent was obtained from patient for publication of this case report and accompanying images.

## Author contributions

KM and KH performed the operation.

KM was responsible for writing this manuscript.

YS, TA, MF, AA, and AI made critical revisions to this article for important intellectual content.

TK gave the final approval of the article.

All authors read and approved the final manuscript.

## Registration of research studies

Not applicable.

## Guarantor

Koichi Mohri.

## Provenance and peer review

Not commissioned, externally peer-reviewed.
